# Latest Approaches to Preventing Alcohol Abuse and Alcoholism

**Published:** 2000

**Keywords:** prevention research, impaired driver, AODR (AOD [alcohol or other drug] related) accident mortality, AOD availability, community-based prevention, AOD advertising impact, econometrics, school-based prevention, AOD laws, trend

## Abstract

Scientists and policymakers have explored numerous strategies to prevent alcohol abuse and dependence as well as the adverse social, legal, and medical consequences of alcohol use. Many of these efforts have focused on reducing alcohol-impaired driving and the associated injuries and fatalities. As reported in this article, such efforts have included general deterrence laws (e.g., reduced minimum legal drinking age, administrative license revocation, and lower legal limits for blood alcohol concentrations), measures targeted at repeat offenders, and measures to control alcohol availability (e.g., increased taxes and decreased numbers of establishments selling alcohol).

In recent years, research on strategies to prevent alcohol abuse and alcoholism has expanded greatly and shifted in emphasis. Whereas studies 10 to 15 years ago focused almost exclusively on education-based prevention approaches, more recent research has evaluated a wider range of prevention measures, including laws and policies to reduce alcohol-related problems at local, State, and national levels. Gauging the effectiveness of such strategies can be complicated by a wide range of factors, such as cultural and economic variability in the study populations or activities at the community or State level that may influence the study outcomes. Using rigorous statistical methods, however, investigators are producing results that can be generalized to populations beyond the study groups, thus offering new understandings of how programs and policies can reduce the toll of alcohol-related problems.

This article reviews the current state of prevention research in several areas. It first explores the results of various efforts to reduce alcohol-impaired driving, such as increasing the minimum legal drinking age (MLDA) to 21 and passing “Zero Tolerance” laws for young drivers. The article then reviews several community-based prevention approaches aimed at reducing alcohol consumption by young people or by all community members. Finally, the article discusses the effects of alcohol advertising on drinking behavior. For a summary of prevention issues related to fetal alcohol syndrome, see the article “Prenatal Exposure to Alcohol” in this issue.

## Reducing Alcohol-Impaired Driving

Alcohol-impaired driving is a major public health problem in the United States. Traffic crashes involving alcohol killed more than 16,000 people in 1997 alone (National Highway Traffic Safety Administration [NHTSA] 1998*b*) and injure a million more each year (Blincoe 1996). Furthermore, 40 percent of fatal traffic crashes, which are the leading cause of death for those aged 1 through 24, involve alcohol (NHTSA 1998*b*; U.S. Department of Health and Human Services 1997). And although annual traffic deaths related to alcohol have dropped by more than one-third since the early 1980s, the dramatic decline in fatalities seen in the early 1990s has leveled off and the number of people killed and injured each year remains staggeringly high.

Several factors have contributed to the drop in alcohol-related traffic fatalities. These include general car safety measures, such as the introduction of air bags and laws mandating the use of seat belts and child restraints, as well as alcohol-specific factors. One likely contributor to the drop in alcohol-related crashes is the reduction in drinking by nearly 20 percent since the early 1980s. The passage of State-level legislation likely also has had an impact. This legislation includes “general deterrence laws” aimed at the population at large as well as “specific deterrence laws” aimed at persons already convicted of alcohol-impaired driving. Active enforcement and education at the community level have been critical to the success of these laws. Reductions in alcohol-related crashes have also resulted from large-scale prevention programs at the community level. Finally, policy measures (e.g., alcohol taxation rates and State monopoly systems) and individual-level initiatives (e.g., personal interventions to prevent alcohol-impaired driving and designated driver strategies) may also reduce impaired driving.

Researchers do not yet know why alcohol-related traffic fatality rates have leveled off since the dramatic drops of the late 1980s and early 1990s. One contributing factor may be a drop in police enforcement, because drunk-driving arrests nationwide have decreased 23 percent since 1983 (Hingson et al. 1996). In addition, questions have been raised as to whether public pressure to reduce drunk driving has dropped in recent years.

**Facts About Alcohol-Impaired Driving**

**How many deaths and injuries?** In 1997 alone, alcohol-related crashes killed more than 16,000 people—an average of one death every 32 minutes (National Highway Traffic Safety Administration [NHTSA] 1998*b*). In addition, an estimated 1 million more people are injured each year in alcohol-related crashes (Blincoe 1996).

**What are the chances?** About 3 out of every 10 Americans will be involved in an alcohol-related traffic crash at some point in their lives (NHTSA 1998*b*).

**Who are the victims?** Alcohol-impaired driving often harms the innocent: In 1996, 40 percent of those killed in crashes involving drinking drivers were people other than the drinking driver. Most of these victims were passengers in the drinking driver’s vehicle (23 percent of all fatalities), followed by occupants of vehicles struck by the drinking driver (12 percent), and pedestrians (5 percent) (NHTSA 1998*a*).

**Who are the drivers?** According to the Behavioral Risk Factor Survey of 102,263 adults ages 18 and older (Liu 1997):

More men than women (4 vs. 1 percent) reported alcohol-impaired driving. The highest rate was reported by males ages 21–34 (7 percent), followed by males ages 18–20 (5 percent).
The highest rate of impaired driving was reported by white males (4.4 percent), compared with 3.1 percent for Hispanic males and 2.8 percent for black males.
Among those who reported “binge” drinking (defined in the study as consuming at least five drinks at a single sitting in the past month), 14.6 percent reported driving while impaired; this rate was thirtyfold higher than that reported by those who did not report binge drinking.

**How many are arrested?** In 1996 alone, 1.5 million people were arrested for driving while intoxicated (NHTSA 1998*b*). Alcohol-impaired driving has been the leading category of arrests over the past decade, accounting for nearly 10 percent of all arrests.

**What are the financial costs?** Alcohol-related traffic deaths and injuries cost the Nation more than $45 billion in lost economic productivity and hospital and rehabilitation costs (Blincoe 1996).

### Recent Trends in Alcohol-Related Traffic Fatalities

The remarkable progress in decreasing alcohol-related traffic fatalities has been documented by National Roadside Surveys conducted in 1973, 1986, and 1996, in which drivers were stopped between 10:00 p.m. and 3:00 a.m. on Friday and Saturday nights, when most drinking occurs (Voas et al. 1997). These surveys revealed the following changes in drinking-and-driving statistics from 1973 through 1996:

- The proportion of drivers with positive blood alcohol concentrations (BACs) dropped 53 percent. The decline was greatest for drivers with BACs between 0.005 and 0.049 percent.
- The proportion of drivers under age 21 with BACs of at least 0.10 percent dropped by 92 percent. This decline may result at least in part from the passage of MLDA laws, which by 1988 made it illegal to sell alcohol to persons under 21 years of age. The proportion of drivers aged 21 through 25 with BACs of at least 0.10 percent dropped by 33 percent.
- The proportion of drivers with positive BACs dropped by 55 percent among white drivers and by 40 percent among black drivers. At the same time, however, the proportion of Hispanic drivers with positive BACs more than doubled.

Data from fatal crashes also confirm the overall declines in alcohol-impaired driving. Between 1982 (the first year that national data were collected) and 1997, alcohol-related traffic fatalities dropped 36 percent. The greatest reductions were among youth aged 15 through 20, whose alcohol-related traffic deaths dropped 59 percent.

### Legislative Efforts To Reduce Alcohol-Impaired Driving

Legislative efforts to reduce alcohol-impaired driving have emphasized laws that deter violations by applying swift, certain, and severe penalties when warranted. Most of this legislative activity has occurred at the State level, although Federal initiatives played a role. Such laws and programs need to deter both first-time and repeat offenses. While people already convicted of “driving under the influence” (DUI) have a higher than average likelihood of further arrests and crashes, most drivers in fatal crashes involving alcohol have never been previously convicted.

#### General Deterrence Laws

Efforts aimed at the general public have focused on the MLDA, zero tolerance laws, administrative license revocation (ALR), and legal limits for BACs. As a result of the 1984 National Minimum Drinking Age Act, all States had enacted a MLDA of 21 by 1988. Numerous studies have demonstrated that increases in the MLDA resulted in decreases in traffic crashes and crash fatalities. Overall, NHTSA (1998*b*) estimates that imposing a MLDA of 21 has prevented more than 17,300 traffic deaths since 1976, or approximately 700 to 1,000 deaths each year for the past decade. MLDA laws also have reduced drinking among people aged 21 through 25 who grew up in States with a MLDA of 21.

An amendment of the National Minimum Drinking Age Act in the fall of 1995 mandated withholding of Federal highway funds from States that did not also adopt laws that make it illegal for those under 21 to drive after drinking any alcohol. As a result, by April 1998, all States had passed “Zero Tolerance” legislation that sets legal BAC limits of zero to 0.02 percent for persons under age 21. The impact of these laws has been significant. The first 12 States to adopt Zero Tolerance laws experienced a 20-percent decline in the proportion of crashes most likely to involve alcohol (i.e., single-vehicle, nighttime fatal crashes) among drivers under 21 compared with 12 nearby States that did not adopt those laws (Hingson et al. 1994).

ALR laws allow a police officer or other official to confiscate a driver’s license immediately if his or her BAC exceeds the legal limit. These laws, which exist in 40 States, permit swift and certain punishment at the time of the infraction, bypassing the court system. One nationwide study found that ALR was associated with a 5-percent decline in fatal crashes and a 9-percent decline in single-vehicle, nighttime fatal crashes (Zador et al. 1989).

All States except Massachusetts and South Carolina have adopted laws that make it a criminal offense to drive with a BAC above the State’s legal limit, which in most States is 0.10 percent. The laws include a provision that the driver’s BAC in and of itself, or “per se,” is enough to demonstrate impairment, so prosecutors do not have to introduce other evidence and thus can make convictions more easily. In addition, 17 States have lowered the legal BAC limit from 0.10 to 0.08 percent.^1^ States that have adopted a 0.08-percent law have experienced significant decreases in alcohol-related fatal crashes. Upon enacting 0.08-percent laws, States can expect, on average, an annual 8-percent decline in the proportion of drivers involved in fatal crashes who have positive BACs (Voas and Tippetts 1999). The lower limits work best when enacted in combination with ALR laws.

#### Laws To Deter Repeat Offenders

Repeat DUI offenders account for approximately one-third of drivers arrested or convicted for DUI each year and for one-sixth of drivers with positive blood alcohol levels who are killed in traffic crashes. Specific deterrence laws seek to reduce this recidivism through a variety of measures, as follows:

- Actions against vehicles and tags, such as license suspensions; use of ignition interlock devices that prevent vehicle operation when a measurement of the driver’s breath alcohol level exceeds a designated limit.
- Lower legal BAC limits for convicted DUI offenders, which have been enacted in Maine. This measure has significantly reduced fatal crashes involving drivers previously convicted of DUI in that State.
- Treatment to rehabilitate DUI offenders. This approach reduces the incidence of repeat offenses by up to 9 percent compared with standard sanctions such as jail or fines.
- Jail sentences, which may have some short-term deterrent effects. However, mandatory jail sentences tend to affect court operations and the correctional process negatively by increasing the demand for jury trials and plea bargains and by crowding jails.
- Mandated attendance of victim impact panels (VIPs)—that is, groups of three or four speakers who were seriously injured or had a loved one killed in a DUI crash and who present their stories to DUI offenders.
- Probation, rather than a jail sentence, which may slightly reduce recidivism among drivers at low risk for being repeat offenders (but not for those at high risk for another DUI citation).
- Detention in facilities maintained specifically for DUI offenders that offer both incarceration and supervised rehabilitation services.

### Enforcement of Impaired-Driving Laws

The extent to which drunk-driving laws are enforced can influence their effect on impaired driving. Drunk-driving arrests increased dramatically between 1978 and 1983 but have dropped since then (NHTSA 1998*b*). The general public may sense this drop in enforcement, because respondents in one survey believed that people who drink and drive are more likely to be in a crash than to be stopped by the police (Jones and Boyle 1996).

Enforcement measures, such as sobriety checkpoints, cannot only enforce laws, but also deter drunk driving. For example, in a California study, the use of sobriety checkpoints reduced alcohol-related crashes regardless of the number of officers present or the number of locations used (Stuster and Blowers 1995). In addition to sobriety checkpoints and arrests of drunk drivers, other legal approaches exist, such as sentencing options that include financial sanctions, publication of offenders’ names in newspapers, victim restitution programs, and court-ordered visits to emergency rooms. The effectiveness of sentencing approaches remains to be determined.

### Alcohol Control Policies

Several laws and policies have attempted to reduce alcohol-related driving deaths by controlling the availability of alcohol as a means of discouraging drinking, particularly among persons under 21. Some of these measures are described in this section.

#### Taxes

Increases in beer taxes have consistently been linked with lower rates of alcohol-related traffic fatalities. One recent study found that for every 1 percent increase in the price of beer, traffic fatality rates could be expected to drop by nearly the same proportion, or 0.9 percent (Ruhm 1996). Higher beer taxes are linked most strongly with lower rates of traffic fatalities that occur at night or among those aged 18 through 20. Increases in alcoholic beverage prices may have little effect, however, on consumption by the heaviest drinkers.

#### Server Training, Sanctions, and Liability

Legally intoxicated drivers are more likely to have just left a bar or restaurant than any other single departure point. Furthermore, breath tests given to patrons leaving bars have indicated that about one-third have BACs above the legal limit. These findings point to a need for server training programs to help waiters, waitresses, and bartenders avoid serving alcohol to people who are already intoxicated. Although evaluations of these training programs have produced mixed results, some studies show that they can modify serving practices to help reduce the amount of alcohol and the rate it is consumed by patrons. As a result of a server training law passed in Oregon in 1985, single-vehicle, nighttime crashes likely to involve alcohol dropped by 23 percent after 3 years. It is difficult to assess, however, whether all of this reduction can be directly attributed to this specific legislation. All States have either criminal or civil sanctions against serving patrons who are obviously intoxicated and most States recognize some form of server liability.^2^

#### State Monopoly Versus Privatized Sales Outlets

Eighteen States have some form of monopoly control over the sale of alcoholic beverages, which influences both the availability and price of alcohol. Compared with States that issue licenses to private retail sellers, in monopoly States spirits are less available, beer is more available, and alcoholic beverages cost more. Relatively little research, however, has examined the effect of State-regulated alcohol sales on alcohol use or related problems.

#### Outlet Density

More than a decade ago, researchers established the connection between the density of outlets in an area and fatal traffic crashes. More recently, investigators reported that regions with greater outlet density and higher ratios of outlets to people had higher alcohol sales (Gruenewald and Ponicki 1995). The study also explored whether a reduced outlet density might lead to increases in fatal crashes as a result of people driving farther to obtain alcohol. However, no such increase occurred.

### Individual Actions

#### Designated Drivers

The use of designated drivers has been widely promoted in the United States since 1988 and is strongly recognized and accepted among the U.S. population. Moreover, in the 1996 National Roadside Survey, most of the designated drivers (82 percent) had BACs between zero and 0.02 percent. In all, about a third of designated drivers consumed some alcohol before driving, but most (95 percent) remained at BACs below the legal limit of 0.08 percent (Fell et al. 1997).

Thus, many people now use designated drivers, and most designated drivers in roadside surveys do not exceed the legal BAC limit. However, designated drivers who do exceed the legal limit, like any driver who does so, are at greater risk of crashing and endangering their passengers.

#### Personal Interventions To Reduce Alcohol-Impaired Driving

Personal interventions (e.g., requests from friends or family members not to drive after drinking), particularly by wives or girl-friends, can have a high degree of success in dissuading impaired people from driving. Furthermore, the older and more sober the person who is intervening, the greater in general is the likelihood of success. Systematic programs to increase personal intervention behavior have not been tested, however, although they clearly warrant consideration.

### Safety Belt Laws

People who drive after heavy drinking and passengers who ride with heavily drinking drivers are less likely to wear safety belts. In fact, legally intoxicated drivers are about one-third less likely to wear seat belts than are other drivers. Although seat belt use clearly saves lives, laws requiring the use of safety belts have not had much impact on traffic injuries and fatalities. This is at least in part because the people most likely to be involved in traffic crashes, such as young males who drive after drinking, have been significantly less responsive to safety belt use laws. Efforts to combine safety belt laws and drunken driving law enforcement should be considered, particularly in “primary” safety belt law States where police can stop motorists simply because they are not wearing safety belts. Such strategies may hold promise both in reducing driving after drinking and increasing safety belt use.

## Community-Based Prevention Approaches

In the last decade or so, researchers, community organizers, and funding agencies have shown a heightened interest in community-based prevention programs. Researchers in the field of alcohol abuse prevention can build upon the technical expertise and practical experience gathered in community-based prevention programs for cardiovascular disease (CVD). These programs have successfully reduced smoking, dietary fat intake, and other risk factors among people who were at high risk of developing CVD. To achieve that goal, CVD programs have emphasized the power of the individual to alter his or her own behavior and employed such strategies as community organization and social marketing tactics to broadcast health messages.

Alcohol researchers face different challenges and opportunities, however, when designing prevention programs. One fundamental difference is that CVD programs aim to alter a medical endpoint (i.e., reducing heart attacks and strokes) by changing people’s behaviors. In contrast, programs on alcohol abuse prevention do not have such a narrowly defined, clinical endpoint. Reducing alcohol-related traffic accidents and other problems associated with drinking, but not necessarily the incidence of drinking, is a legitimate goal. In addition, researchers in alcohol prevention programs must take into consideration that excess alcohol consumption can lead to trauma far more quickly than excess fat consumption or other risk factors can lead to CVD. Finally, the fairly complicated social standards associated with drinking generally do not exist with health habits that contribute to CVD. Alcohol researchers, however, have the advantage of many ready-made opportunities to develop prevention efforts by tapping into local regulatory systems that address alcohol safety issues.

### Recent Research Results

Three major community-based studies to prevent alcohol problems have been conducted in the last 3 years. These studies—Project Northland, Communities Mobilizing for Change on Alcohol, and the Community Trials Project—are described in this section.

#### Project Northland

A school-based program aimed at preventing the onset of adolescent drinking, Project Northland used a broad approach that considered environmental influences, in particular the effects of peers. The project focused on high-risk, relatively heavily drinking communities in northern Minnesota, surveying students who were in sixth grade and tracking them for 3 years (Perry et al. 1996). The researchers formed a sample of 20 sites, then randomly assigned 10 sites to the intervention, or treatment program. The other 10 sites, which served as a comparison set, continued whatever programs they had in place.

The project team developed three different treatment programs, one each for grades six through eight. Each program included components not only for students, but also for parents and the larger community. From grades six through eight, the materials and activities moved from a focus on the self to interactions with parents, peers, and the community at large, thus engaging the students in expanding “concentric circles of influence.”

The researchers surveyed 2,350 students at the beginning of the study, then conducted follow-up surveys at the end of each of the 3 school years. The surveys focused primarily on the use of alcohol (past month and past week), tobacco (cigarettes and smokeless tobacco), and marijuana. The researchers also assessed the “tendency to use alcohol”^3^ and measured various other factors (e.g., peer influences).

By the eighth grade, students in the treatment sites had lower rates of alcohol use, both in the past month and past week, as well as lower scores on the “tendency to use alcohol” scale. These statistically significant differences were observed despite the fact that the intervention sites, though selected at random, had higher alcohol use rates at the beginning of the study. No significant differences were observed for cigarette, smokeless tobacco, or marijuana use—which were not targets of the program—except for a higher initial rate for cigarette use in the intervention sites. On the measure of general peer influence, students in the intervention sites had lower scores in the eighth grade than did those in the comparison sites (at the start of the study there were no significant differences between the sites). No significant differences were observed between the treatment and comparison schools for any other factors. Overall, the program appeared to affect those students who had not yet begun to use alcohol, but had little or no impact on those students who were already drinking.

#### Communities Mobilizing for Change on Alcohol (CMCA)

This community-organizing effort was designed to reduce drinking and drinking-related problems among 15- to 20-year-olds by reducing their access to alcohol. It aimed to build the capacity of communities to change their own policies and practices related to alcohol access (Wagenaar et al. 1994). The study focused on five areas: (1) influences on community policies and practices and on parents and others to support these efforts; (2) the policies and practices themselves, in particular formal laws against serving minors and informal practices related to enforcing those laws; (3) youth access to alcohol; (4) youth alcohol consumption; and (5) youth alcohol problems. The study included 15 communities in Minnesota and western Wisconsin, each of which had a school district with at least 200 ninth graders. The researchers formed seven sets of two or three communities, with each set having one randomly selected intervention site and one or two comparison sites.

The researchers sought to organize the communities to develop their own specific interventions to influence under-age access to alcohol. Each community could include any number of activities, such as using decoy operations in which underage buyers attempt to purchase alcohol at selected outlets, citizen monitoring of outlets selling to youth, requiring keg registration, developing alcohol-free events for youth, shortening hours of sale for alcohol, initiating responsible beverage service (RBS) training, and developing educational programs for youth and adults. The researchers gathered evaluation data before the interventions began and again 2.5 years later, focusing on questions about alcohol use, purchasing behavior, perceptions of availability, and other measures.

The results of the intervention were mixed. After the 2.5-year program, merchants checked more frequently for age identification, reduced their likelihood of sales to minors, and reported more care in controlling sales to youth. Among 18- to 20-year-olds, the frequency of providing alcohol to other minors as well as the likelihood of buying and consuming alcoholic beverages themselves was reduced. Although these results were not, in themselves, statistically significant, consistent progress was made in all seven intervention sites.

#### The Community Trials Project (CTP)

This program differed from the previous two studies in two important respects. It aimed to reduce injuries and deaths related to drinking rather than alcohol consumption itself, and it targeted the entire population rather than adolescents only (Holder et al. 1997; Treno and Holder 1997). The project was conducted in three areas—northern California, southern California, and South Carolina—each containing one experimental community matched with one comparison site. The program consisted of five interacting project components, as follows:

- *Community knowledge, values, and mobilization*. This component aimed to help mobilize the communities to develop and implement their own action plans for addressing local problems. As the communities mobilized, broad-based coalitions provided general support for project goals, while task forces developed specific strategies and intervention plans. The researchers identified six points as essential in mobilizing communities to support prevention programs (see textbox above).
- *RBS practices.* This component was aimed at reducing the likelihood of customer intoxication at licensed bars and restaurants by implementing RBS practices, such as monitoring customer’s alcohol consumption, preventing intoxicated patrons from driving, serving drinks in standard serving sizes, promoting consumption of food and nonalcoholic beverages, and avoiding price promotions for alcoholic beverages. Although RBS training can improve server practices in a given establishment, voluntary training in only a portion of a community’s establishments had little effect on the community as whole.
- *Reduction of underage drinking.* This component used three intervention strategies—enforcement of underage sales laws, clerk training and outlet policy development, and media advocacy. After the intervention, experimental community outlets were about half as likely as those were in comparison sites to sell alcohol to an apparent minor.
- *Risk of drinking and driving.* This component also used three intervention strategies—expansion of DUI news coverage, implementation of sobriety checkpoints, and use of a special breath-testing program. The activities of this component had a statistically significant impact upon traffic crashes involving alcohol, resulting in an annual reduction of 10 percent.
- *Access to alcohol.* The final component used three intervention strategies as well—mapping and publicizing the connections between the geographic availability of alcohol and alcohol-related problems, developing local planning and zoning policies to regulate the density of alcohol outlets, and encouraging community action to revoke licenses from problem outlets. Ultimately this component led to greater local input in license renewal; new regulations regarding special event permits, such as banning alcohol at some public activities; and successful protests of licenses that eliminated sales of alcohol from problem outlets.

**Factors Essential for Mobilizing Communities to Support Prevention Programs**

- Explain the research base for the interventions to the community participants.
- Maintain existing community coalitions and require project staff guidance only when considering specific interventions.
- Use pre-existing community support for rapid implementation of project interventions.
- Use existing support among community leaders to focus mobilizing efforts.
- Use community events (e.g., local festivals) as opportunities to initiate interventions and galvanize public support.
- Employ media events to help generate project enthusiasm.

### Commentary and Future Research Needs

Although none of these community-based alcohol prevention studies has reported substantially large impacts on their chosen targets, a real effect may be seen in all of them. Each study was predicated on the belief that with a community-wide effort, the final outcome would be greater than merely a sum of the individual parts. However, individual aspects of these studies were so well developed that the observed impact might have been achieved even without the benefit of other community activities.

The extent to which the outcome of these community studies can be generalized is still unknown. A particular impact demonstrated by a study may not be detected in other settings where implementation may be less intense, where evaluation data may not be available, or where the program does not benefit from a “halo” derived from academic involvement. Thus, neither the costs nor the benefits of conducting an intervention in one community will necessarily transfer to other communities. A related concern is how to sustain the impact of community interventions, which may depend on large infusions of resources, or fade in time as the novelty wears off.

## Alcohol Advertising: What Are the Effects?

In recent years, public health advocates have called for strict regulation or elimination of alcohol advertising, and community-level action has focused on reducing local alcohol advertising. Does alcohol advertising increase the overall level of alcohol consumption? Does it predispose children and adolescents to drinking? Although these and other related questions have been raised by public health advocates and echoed in public opinion surveys, the evidence from research to date is mixed and far from conclusive. In general, studies based on economic analyses suggest that advertising does not increase overall consumption, but instead may encourage people to switch beverage brands or types. At the same time, research based on survey data indicates that children who like alcohol advertisements intend to drink more frequently as adults. While these findings might offer some grounds for both reassurance and concern, the limitations of the research methods that have been used hinder the ability to draw firm conclusions about cause and effect in either case.

The concerns about alcohol advertising stem at least in part from its pervasiveness. The alcohol industry spent $1.03 billion on alcohol advertising in 1996, with the expenditures concentrated on television commercials and beer advertising (Besen 1997). Overall, alcohol commercials make up 1.5 percent of all advertisements on prime-time television and 7.0 percent of all advertisements in sports programming. In addition, alcohol advertisers use other types of promotions (e.g., stadium signs, sponsorship of television programs, and on-site promotions) embedded in sports programming to promote their products to the television viewing audience.

Researchers have been particularly interested in the degree to which children and adolescents pay attention to these commercials. In one survey of fifth- and sixth-grade children, 59 percent of the children could correctly identify the brand of beer being promoted from an edited, still photograph taken from a television commercial (Grube 1995). Alcohol advertising with celebrity endorsers, humor, animation, and rock music is especially appealing to adolescents (Atkin and Block 1980; Grube 1995).

Researchers have employed four main types of studies to investigate the effects of advertising: experimental research in controlled settings; econometric analyses, which apply economic research techniques; surveys; and intervention studies of “media literacy” programs that encourage skepticism about advertisements. The results of such studies are described in the following sections.

### Experimental Studies

Experimental studies have investigated how short-term exposure to alcohol advertising affects people’s drinking beliefs and behaviors under controlled conditions. Typically, a group of participants is exposed to one or more alcohol advertisements embedded among a series of neutral advertisements. The investigators then compare the experimental group’s beliefs or behaviors related to drinking with those of a control group that views the same items without the embedded alcohol advertisements. This approach has yielded mixed results, with some studies finding no effects and others finding small or short-term effects for some study participants.

One study applied this approach to examine the effects of television beer advertising on the drinking beliefs of young people who were not regular drinkers (Lipsitz et al. 1993). The study found that neither exposure to beer advertisements nor to antidrinking public service announcements affected the children’s expectancies about the outcomes of drinking.

Overall, the results of experimental studies offer only limited support, at best, for the assumption that alcohol advertising affects drinking beliefs. This may in part be due to the lack of realism inherent in laboratory studies. Furthermore, the stimulus advertisements may not contain images, themes, or music that appeal to the participants in a specific study, reducing the likelihood of observing any effects. Finally, laboratory experiments can only address the effects of short-term exposure to a limited number of alcohol advertisements. The relevance of such studies for understanding the cumulative effects of exposure to hundreds or thousands of alcohol advertisements over many years is questionable.

### Econometric Studies

A number of studies have applied the theoretical and statistical techniques of economic research to analyze issues relating to alcoholic beverage advertising. Generally these econometric studies have focused on the relationship between the advertising expenditures of the alcohol industry and the average amount of alcohol consumed per person (i.e., per capita consumption) or the amount of alcohol sales, with price and other factors taken into account. A few studies have investigated whether alcohol advertising affects rates of traffic fatalities and other alcohol-related problems such as liver cirrhosis. Overall, the econometric studies conducted to date provide little consistent support for a relationship between alcohol advertising and alcohol consumption and related problems. They do provide indirect support, however, for the hypothesis that alcohol advertising leads to changes in brand or beverage preferences without increasing total consumption.

#### Effects on Alcohol Consumption and Alcohol-Related Problems

Econometric studies conducted prior to 1990 generally concluded that alcohol advertising exerts a negligible effect on *overall alcohol consumption*. Two more recent studies, in contrast, reported substantive and statistically significant effects of alcohol advertising on *alcohol-related problems*. One of those studies (Saffer 1997) examined the relationship between motor vehicle fatalities and variations in local alcohol advertising in the top 75 media markets in the United States from 1986 through 1989. The investigator found that increases in alcohol advertising were significantly related to increases in total and nighttime vehicle fatalities. The effects appeared to be greater for older drivers than for younger drivers (18 through 20 years old). Although the study cannot firmly establish a causal relationship, it offers the strongest econometric evidence to date that alcohol advertising might influence drinking problems. The divergence of the findings of this study from some earlier econometric studies may, in part, be a result of improvements in methodology.

#### Reallocation of Market Shares

Several other recent econometric studies analyzed the association between advertising and per capita consumption of specific brands or types of alcoholic beverages. In addition, they looked for “cross-sectional associations,” or links between consumption and advertising in specific, narrow, time frames. Overall, while providing little or no evidence that alcohol advertising increases overall alcohol consumption, the findings of several studies suggest that such advertising may realign market shares among brands and, to a lesser extent, among different beverage types.

One limitation of econometric studies is that they tend to combine, or aggregate, the advertising data across the different media types, which prevents researchers from detecting the separate effects of each type. In addition, the use of data aggregated yearly may hide the short-term effects of “pulsed” advertising campaigns that have peaks and valleys in the concentration of advertisements over the year. Furthermore, existing econometric studies have focused on per capita consumption, problems, or sales rather than on the drinking behaviors of individual drinkers. Thus, the finding that alcohol advertising has no aggregate effect on consumption does not mean that there is no effect for any individual drinker. Not enough is known about how alcohol advertising might affect specific populations that may be more susceptible or more exposed to the advertising, such as young people or minorities.

### Survey Studies

For the most part, survey studies of alcohol advertising have focused on children and adolescents. Many of those studies have found small but significant positive relationships between exposure to, or awareness of, alcohol advertising and drinking beliefs and behaviors among young people. For example, in one study the investigators found that the children who were more aware of advertising had increased knowledge of beer brands and slogans as well as more positive beliefs about drinking. In addition, those with higher levels of awareness of alcohol advertising were slightly more likely to say that they intended to drink as an adult (Grube 1995; Grube and Wallack 1994). Based on their analyses, which accounted for the children’s prior beliefs and knowledge, the researchers suggested that awareness of alcohol advertising predisposes young people to drink, rather than the other way around.

Several studies have attempted to find out whether children and adolescents who like alcohol advertisements have different drinking beliefs and behaviors than those who do not like the advertisements. These studies found that children and adolescents who liked alcohol advertisements more were more likely to have experimented with alcohol; have positive expectations regarding drinking; expect to drink more frequently at age 20; drink at greater rates; agree with positive belief statements about alcohol; and report drinking problems, such as physical fights.

Although survey studies consistently find significant associations between alcohol advertising and drinking beliefs and behaviors, these relationships only tend to be modest. Moreover, because of the cross-sectional designs of most of these studies, the results allow no conclusions about causality (i.e., whether favorable views of advertisements predispose to drinking or vice versa).

### Media Literacy Interventions

Many school-based education programs involve “media literacy” or “resistance to social influence” curricula designed to increase students’ ability to think analytically about advertising. One recent study assessed the effects of providing general or alcohol-specific media literacy education to young children (i.e., third graders) (Austin and Johnson 1997). The children viewed either a “general media education” video designed to promote skepticism toward advertising, an “alcohol-specific media education video,” or neither video. The intervention had both immediate and delayed effects. Immediately after the intervention, the children who had received media literacy education showed a decrease in their desire to be like the actors portrayed in advertisements and viewed these actors as less similar to them and as less desirable. They also showed a decrease in their expectations about the positive effects of drinking and were less likely to select alcohol-related products and toys when given a choice.^4^ These intervention effects were maintained after 3 months. The analyses also indicated that the alcohol-specific intervention was more effective on these measures than the general media literacy intervention, and that the intervention influenced girls more than it did boys.

This and other studies suggest that exposure to alcohol-specific media literacy education may increase resistance to alcohol advertising months or even years later. The ability to generalize the results of these studies to a broad population is limited, however, because the studies used samples that were not representative of the population at large. Furthermore, the long-term effects of these childhood interventions on actual drinking behavior (e.g., initiation of drinking or drinking frequency in later adolescence) remain unknown.

## Summary

As a result of various prevention measures, alcohol-related traffic deaths have declined remarkably since 1982. Progress has slowed in recent years, however, and the current level of 16,000 deaths and more than 1 million injuries in alcohol-related traffic accidents each year demonstrates the need for continuing attention to this major public health problem. Further reductions could be achieved if all States adopted ALR; Zero Tolerance laws for youth; 0.08-percent “criminal per se” laws for adults; and mandatory treatment, if needed, for convicted DUI offenders. These laws would have the greatest benefits if they were actively publicized and enforced at the community level through checkpoints and comprehensive community programs. Stimulating public concern and developing new ways to engage private citizens to work with local government departments will be key challenges for the next decade.

Studies of community-based prevention programs, such as Project Northland, CMCA, and CTP, have provided some evidence that they can help prevent alcohol problems. Although community-based prevention research is in its early development, interest in full-scale community studies should be complemented with a range of smaller studies to allow better identification of potentially powerful interventions and the best ways of implementing them. This approach would require peer reviewers, policymakers, and funding agencies to recognize the value of such modest studies even where a “final” answer regarding impact is not forthcoming.

The results of research on the effects of alcohol advertising are mixed and not conclusive. Overall, experimental studies have produced little consistent evidence that alcohol advertising affects drinking beliefs and behaviors. By their nature, however, these studies examine short-term exposure to a limited number of advertisements and thus do not provide insight into the cumulative effects of exposure to alcohol advertising over many years. Large-scale field experiments that block alcohol advertising from reaching selected communities or households could provide stronger evidence about the effects of alcohol advertising.

Similarly, recent econometric research using aggregated market data provides very little consistent evidence that alcohol advertising influences per capita alcohol consumption, sales, or problems. The bulk of this research supports the claim that alcohol advertising reallocates consumption among brands or beverage types.

In contrast to experimental and econometric studies, survey research on alcohol advertising and young people consistently indicates small but significant connections between exposure to, and awareness of alcohol advertising, and drinking beliefs and behaviors. The evidence, however, is far from conclusive. Longitudinal or sequential studies that track samples of young people from childhood to late adolescence, and that control adequately for past drinking behaviors and predisposition, would be particularly useful in investigating whether awareness of alcohol advertising predisposes young people to drink or vice versa.

## Footnotes

1 Massachusetts has set the BAC for its ALR law at 0.08 percent.

2 Server liability means that people can sue for damages incurred as a result of service to a minor or intoxicated patron.

3 The “tendency to use alcohol” was determined by creating a scale that combined certain survey responses, such as linking a student’s actual use of alcohol with his or her intention to use it.

4 The choice between equivalent alcohol- and non-alcohol-related toys serves as a measure of “predrinking” behavior in young children.
